# Research Progress on the Anti-Aging Potential of the Active Components of Ginseng

**DOI:** 10.3390/nu15153286

**Published:** 2023-07-25

**Authors:** Jingqian Su, Qiaofen Su, Shan Hu, Xinglin Ruan, Songying Ouyang

**Affiliations:** 1 Fujian Key Laboratory of Innate Immune Biology, Biomedical Research Center of South China, College of Life Sciences, Fujian Normal University, Fuzhou 350117, China; 15260328242@163.com (Q.S.); qbx20220152@yjs.fjnu.edu.cn (S.H.); 2 Provincial University Key Laboratory of Microbial Pathogenesis and Interventions, College of Life Sciences, Fujian Normal University, Fuzhou 350117, China; 3 Department of Neurology, Fujian Medical University Union Hospital, Fuzhou 350001, China; xlruan@163.com; 4 Key Laboratory of OptoElectronic Science and Technology for Medicine of the Ministry of Education, Fujian Normal University, Fuzhou 350117, China

**Keywords:** ginseng, aging, DNA damage, DNA repair

## Abstract

Aging is a cellular state characterized by a permanent cessation of cell division and evasion of apoptosis. DNA damage, metabolic dysfunction, telomere damage, and mitochondrial dysfunction are the main factors associated with senescence. Aging increases β-galactosidase activity, enhances cell spreading, and induces Lamin B1 loss, which further accelerate the aging process. It is associated with a variety of diseases, such as Alzheimer’s disease, Parkinson’s, type 2 diabetes, and chronic inflammation. Ginseng is a traditional Chinese medicine with anti-aging effects. The active components of ginseng, including saponins, polysaccharides, and active peptides, have antioxidant, anti-apoptotic, neuroprotective, and age-delaying effects. DNA damage is the main factor associated with aging, and the mechanism through which the active ingredients of ginseng reduce DNA damage and delay aging has not been comprehensively described. This review focuses on the anti-aging mechanisms of the active ingredients of ginseng. Furthermore, it broadens the scope of ideas for further research on natural products and aging.

## 1. Introduction

Aging is an inevitable process that affects all individuals. The process is marked by a gradual decline in mobility and metabolic quality, accompanied by phenotypic changes in cellular characteristics, including cell growth arrest [1], chromatin remodeling, metabolic reprogramming, impaired autophagy, and the secretion of pro-inflammatory factors [2,3,4]. DNA damage, metabolic dysfunction, telomere damage, and mitochondrial dysfunction are the main causes of aging [5]. DNA damage is a major driver of aging; it induces permanent cell cycle arrest, and the associated markers accumulate in senescent cells with age [6].

Ginseng (*Panax ginseng C. A. Meyer*) is a perennial plant and a valuable medicinal herb that belongs to the Araliaceae family. Its main components include ginsenosides, polysaccharides, amino acids, volatile oil, and polyacetylene. Historically, ginseng is known as the “king of herbs” and has been widely used to treat various diseases. For example, it was used to slow down the aging process through DNA protection achieved by the reduction in oxidative stress and regulation of intestinal microorganisms [7,8,9,10,11].

This review highlights the active components of ginseng that can delay aging; the relationship between ginseng, DNA damage, and aging; and the anti-aging mechanism of the active components.

## 2. Aging Process

DNA damage is a major cause of aging, which is triggered by chromosomal telomere shortening. Therefore, cell cycle arrest is an important mechanism associated with the progression of aging [6]. Under physiological conditions, DNA is susceptible to attacks from extracellular forces or intracellular metabolites; these attacks can lead to various forms of DNA damage, including the formation of an apurinic/apyrimidinic (AP) site; the oxidation, nitrosylation, and alkylation of DNA bases; single-strand and double-strand breaks; and other modifications [12]. Responses to DNA damage include DNA damage recognition, checkpoint activation, cell cycle arrest, and ultimately, DNA repair, apoptosis, or senescence [13]. The main phenotypes observed in senescent cells include increased lysosomal β-galactosidase activity, enhanced cell spreading, telomere shortening, and loss of laminin B1 [14]. In addition, senescent cells produce senescence-associated secretory phenotypes (SASPs) that are mainly cytokines, chemokines, growth factor proteases, and metalloproteinases [4]. These factors act in both paracrine and autocrine manners [15] to accelerate tissue and aging. The process leads to the manifestation of local and systemic pathological features and the increased incidence of age-related diseases, such as osteoarthritis, atherosclerosis, type 2 diabetes [16,17,18], and neurodegenerative diseases [19,20].

**Figure 1:**
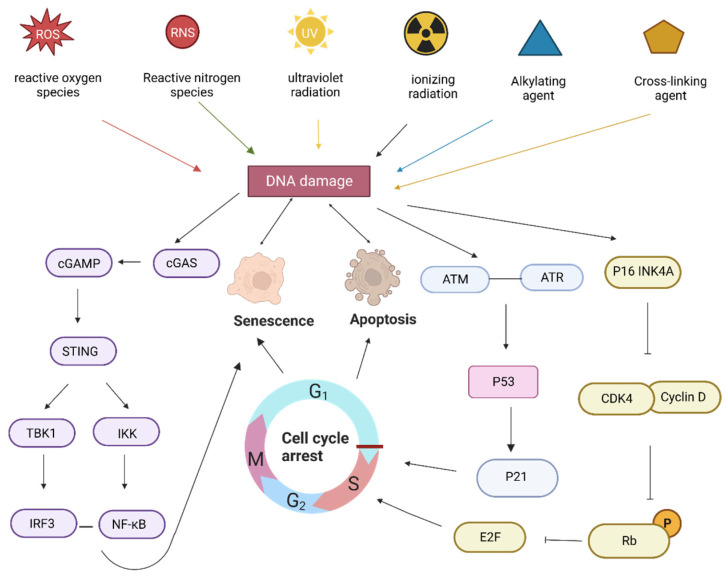
Pathway of DNA-damage-induced aging.

As shown in Figure 1, sustained DNA damage activates DNA damage response pathways, including the ataxia telangiectasia-mutated gene (ATM), Rad3-related gene (ATR), and p53 [21] pathways. DNA damage triggers the activation of the cell-cycle-dependent protein kinase inhibitor, p21, which promotes cell cycle arrest and induces senescence. In addition, DNA damage activates P16INK4a, a cell-cycle-dependent protein kinase inhibitory protein that inhibits CDK4 binding to cell cycle protein D [2]. This prevents retinoblastoma (RB) phosphorylation, and the hypophosphorylated state of RB leads to the inhibition of E2F-dependent gene expression and the blockade of G1/S cell cycle progression, which causes cell cycle arrest and ultimately induces senescence [4,22].

**Figure 2:**
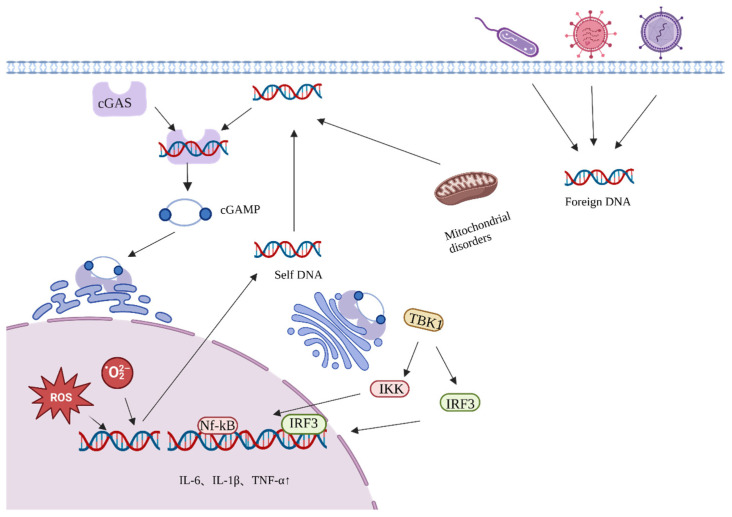
Activation process of the cGAS-STING pathway.

In addition, the transfer of free DNA from the nucleus or mitochondria to the cytoplasm can promote inflammation and accelerate aging [23,24]. Cyclic guanosine monophosphate–adenosine monophosphate (GMP-AMP) synthase (cCGAS) recognizes bound DNA in the cytoplasm and induces a conformational change in the catalytic center of cGAS to convert guanosine triphosphate (GTP) and adenosine triphosphate (ATP) to GMP-AMP (cGAMP) (Figure 2) [25]. cGAMP serves as a second messenger, inducing a conformational change in STING, which is then transferred from the endoplasmic reticulum to the Golgi apparatus. STING recruits and activates TANK-binding kinase 1 (TBK1) and IFN regulatory factor 3 (IRF3), respectively, through a phosphorylation-dependent mechanism. This triggers the activation of NF-κB and initiates the transcriptional induction of downstream pro-inflammatory cytokine genes associated with senescence.

## 3. Anti-Aging Properties of the Active Components of Ginseng

The active ingredients of ginseng can be categorized into saponins, polysaccharides, amino acids, volatile oils, polyethylenes, and other substances. Polysaccharides are mainly amylose glucan and pectin [26,27]. The main amino acids are arginine, glutamic acid, and aspartic acid [28]. Volatile oils mainly include aldehydes, heterocycles, sesquiterpenes, fatty acids, fatty acid esters, and alkanes, of which, sesquiterpenes are the most abundant [29]. Polyacetylenes are mainly diacetyl alcohol, triacetyl alcohol, acetic acid, and linolenic acid [30,31]. In addition, salicylamine, maltose, glucoside, vitamins, enzymes, and various trace components have been isolated and identified from ginseng [32,33]. The purified compounds and extracts derived from ginseng possess the potential to be utilized in various manners for the purpose of retarding the aging process, as indicated in Table 1 and Table 2. Ginsenosides can slow down the aging process by regulating the immune system, mitigating DNA damage through antioxidant and anti-inflammatory mechanisms, and protecting the nervous system [34,35,36,37,38]. Ginseng volatile oil has been shown to prolong the life span of experimental animal models, including *Drosophila* and *Caenorhabditis elegans*, owing to its antioxidant and anti-aging effects [39]. Bioactive peptides reduce the content of senescence markers in NIH/3T3 mouse fibroblasts, significantly inhibit S-phase cell cycle arrest, promote DNA synthesis, and delay cellular senescence [28].

## 4. Anti-Aging Mechanism of Ginseng

### 4.1. Active Ingredients of Ginseng Delay Aging by Reducing Endogenous Oxidative DNA Damage

**Figure 3:**
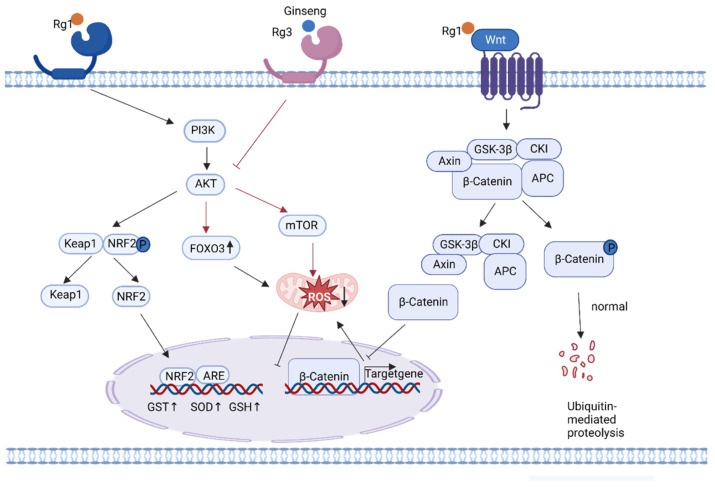
Pathway of ginseng active ingredients reducing endogenous DNA damage.

DNA damage is mainly divided into two types: spontaneous endogenous damage caused by intrinsic factors within the organism (such as reactive oxygen species (ROS) and cellular metabolic byproducts) and exogenous damage caused by the external environment (such as ionizing radiation and chemicals) [57,58]. Intracellular ROS is the most common factor associated with endogenous damage. The mitochondrial respiratory chain is one of the main sources of ROS in cells. Intracellular mitochondrial dysfunction or endoplasmic reticulum stress can lead to ROS production [59]. ROS oxidize nucleoside bases, attack the double bonds of DNA molecules, and induce single- or double-stranded DNA breaks [60,61]. Excessive ROS production leads to intracellular oxidative/antioxidative dysregulation that causes oxidative stress and further DNA damage [62]. Therefore, a reduction in the excessive production of ROS can achieve a balance between intracellular oxidation and antioxidation and ultimately alleviate oxidative stress, which is an important strategy for delayed aging. The active components of ginseng, Rg1, Rg3, and Re, increase the expression of antioxidant enzymes by regulating the dissociation of Kaep1 and Nrf2 proteins in the Keap1/Nrf2/ARE pathway, inhibiting the expression of mammalian target of rapamycin (mTOR) proteins in the Akt-mTOR pathway, and activating the Wnt/β-linked protein signaling pathway. These reduce ROS production [8,36,38], improve oxidation/antioxidation balance, mitigate endogenous DNA damage, and ultimately slow down aging (Figure 3). 

PI3K/Akt/Nrf2 signaling plays a central role in aging-related diseases [63]. Nrf2 is a key redox-sensitive transcription factor that regulates antioxidant defense in various cells by protecting against endogenous and exogenous oxidative stress [64], increasing the activity of antioxidant enzymes, and maintaining normal mitochondrial function and structure [65]. Kelch-1ike ECH-associated protein l (Keap1) is an oxidative stress sensor that normally binds to Nrf2 in the cytoplasm to form a complex [66]. Nrf2 phosphorylation promotes the dissociation of Nrf2 from Keap1 in response to oxidative stress within the organism (Figure 3). Nrf2 translocates to the nucleus and binds to the au-rich element to promote the expression of various downstream antioxidant enzymes, such as glutathione S-transferase, glutathione, and superoxide dismutase, to exert antioxidant capacity and ultimately maintain the oxidative/antioxidant balance and reduce DNA damage [67]. Ginsenoside Rg1 promotes the expression of antioxidant enzymes by activating the PI3K/Akt pathway and phosphorylating Nrf2 [40]. Forkhead box protein O transcription factor 3 (FoxO3) is a longevity gene [68]. Klotho, one of the first anti-aging genes to be identified, increases FoxO3 activity and suppresses ROS-related oxidative stress by inhibiting the activities of phosphatidylinositol 3 kinase (PI3K) and serine-threonine kinase Akt (AKT) [69]. Ginseng and Klotho share the same effect. Ginseng enhances the activity of FoxO3a by inhibiting PI3K and AKT, which results in the reduction in mitochondrial damage, the maintenance of mitochondrial morphology, the reduction in ROS production, and the regulation of aging-related traits [70].

In addition, mTOR is a key serine-threonine protein kinase located downstream of the PI3K/Akt signaling pathway; mTOR overactivation leads to increased ROS production and increases the likelihood of endogenous oxidative damage [71]. Ginseng and ginsenoside 20(S)Rg3 can inhibit PI3K/Akt to downregulate mTOR expression, reduce ROS production, and mitigate oxidative stress-induced aging [71,72]. *Panax ginseng* saponins protect chondrocytes from senescence and apoptosis by downregulating PI3K/Akt/mTOR phosphorylation, preventing a decline in mitochondrial membrane potential, regulating mitochondrial permeability to maintain normal mitochondrial morphology, and reducing ROS production [73].

The activation of the classical Wnt/β-linked protein signaling pathway reduces ROS production [74]. The Wnt/β-catenin signaling pathway is mainly composed of β-catenin, glycogen synthase kinase-3 (GSK-3), casein kinase 1 (CK1), APC, Axin, and the β-catenin complex (Figure 3). Under normal conditions, β-catenin is phosphorylated by GSK-3β and CK1, and the phosphorylated β-catenin is targeted for ubiquitination and degradation. Oxidative stress results in the inactivation of GSK-3β through phosphorylation; consequently, β-catenin is transferred to the nucleus, facilitating the progression of transcription. Ginsenoside Rg1 promoted β-catenin degradation, inhibited β-catenin expression, reduced oxidative stress, and alleviated age-related neurological disorders in mice by increasing GSK-3β phosphorylation [75,76].

### 4.2. Ginseng Active Ingredients Delay Aging by Reducing Exogenous Oxidative DNA Damage

Exogenous DNA damage is mainly triggered by ionizing radiation (IR), cosmic radiation, ultraviolet (UV) radiation, and chemicals in the external environment [77,78]. Radiation acts in a direct way by applying the released energy to biological macromolecules, causing DNA breaks [79]. Indirect molecule stimulation in substances leads to high free radical and ROS production, damaging biomolecules, causing oxidative DNA damage, dysregulating cellular signaling pathways, and inducing aging [78]. The skin is the largest organ of the body and a common site for exogenous injury. Matrix metalloproteinases (MMPs) remodel the extracellular matrix and can degrade collagen [80]. UV exposure leads to MMP-1 upregulation, which destroys collagen fibers, allowing them to be further degraded by other members of the MMP family, leading to skin aging [81,82,83]. The active ingredients of ginseng mainly downregulate MMP via signaling pathways such as MAPK/ERK/p38/JNK, MITF, and P53 to initiate anti-photo-aging, anti-wrinkle, and anti-melanin production to slow exogenous damage-induced aging caused by exogenous damages such as UV radiation [84,85,86].

**Figure 4:**
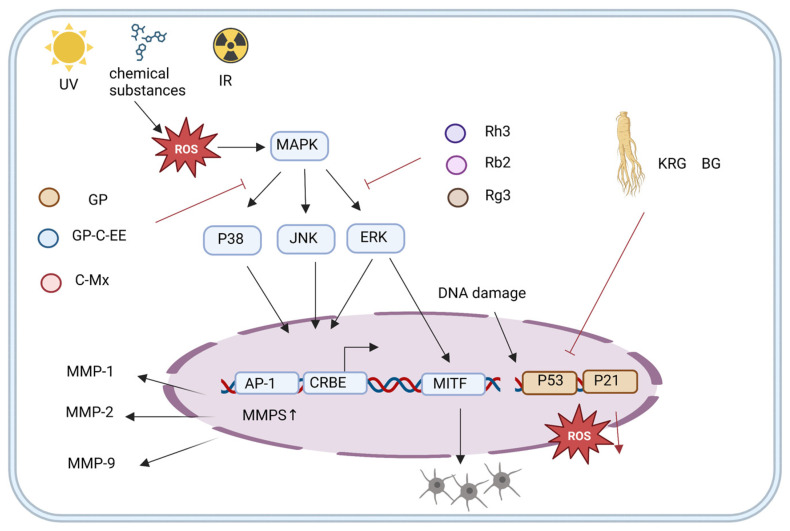
Ginseng active ingredient pathway of reducing exogenous DNA damage.

The MAPK signaling pathway, which includes the MAPK kinase kinase (MKKK), MAPK kinase (MKK), and MAPK components, responds to both extracellular and intracellular signals to influence the cell fate [87]. AP-1 is an intracellular transcriptional activator that regulates MMP expression and catalyzes dermal collagen degradation. When UVB stimulates cells to produce large amounts of ROS, ginseng protein (GP) and ginseng calyx ethanol extract (Pg-C-EE) can inhibit the ERK, p38, and JNK expression; block AP-1 and CRBE transcription; reduce MMPS production; and slow skin aging (Figure 4) [88,89]. Furthermore, C-Mx, an active ingredient derivative of ginsenoside, also demonstrates a certain anti-aging ability. In addition to inhibiting ERK, p38 JNK, and AP-1 expression, C-Mx also promotes procollagen synthesis by regulating the TGF-β/Smad pathway, maintains the cellular oxidative/antioxidative balance, reduces exogenous oxidative damage, and alleviates skin aging [90].

When cells feel stimulated by UV radiation from an external source, they generate a large amount of melanin so that they absorb UV radiation from the outside world, which in the long run will lead to abnormal pigmentation and induce skin aging [91,92]. MITF is a basic helix–loop–helix/leucine-zipper transcription factor essential for melanocyte development and survival and controls melanocyte proliferation [93]. Ginsenoside Rh3, Rb2, and Rg3 (multiple active ingredients) inhibit ERK expression, preventing the transcription factor MITF from exercising its function, inhibiting excessive melanin production, preventing age spot formation, and slowing skin aging [94,95,96]. In addition, when photo-oxidation occurs with UVB radiation, MMP upregulation induces superoxide radical generation, which are converted to ROS, hydrogen peroxide, and other compounds [97]. Rb3, an active ginseng ingredient, slows skin photoaging by downregulating Pro-MMP2 and Pro-MMP3 expression, increasing GSH levels, and decreasing UVB-induced ROS levels to reduce the exogenous DNA oxidative damage [98].

In addition to reducing exogenous DNA damage via the MAPK/ERK/p38/JNK and MITF pathways, ginsenosides can also slow aging via p53, a multifunctional protein involved in DNA repair, metabolic pathway control, embryo implantation, and the driving force of cellular senescence [99]. Koryo Red Ginseng (KRG) extract repairs DNA damage via P53 signaling regulation, inhibits radiation-induced apoptosis, and prevents intracellular ROS production in HaCaT cells [100]. Black ginseng (BG) delays senescence induced by exogenous factors such as ionizing radiation in primary murine embryonic fibroblasts via P53-P21 protein downregulation [54].

### 4.3. Active Ingredients of Ginseng Slow Down Aging by Regulating DNA Damage Repair

At least five major DNA repair pathways are associated with DNA damage in organisms: base excision repair (BER), nucleotide excision repair, mismatch repair, homologous recombination (HR), and non-homologous end joining (NHEJ) [62]. DNA damage that has not been accurately repaired can lead to genomic rearrangements and transcriptional dysregulation, contributing to cellular senescence, apoptosis, or uncontrolled division [57,101,102]. p53 is involved in DNA repair, metabolic pathway control, and cellular senescence. Its main function is to induce apoptosis and cell cycle arrest [99]. p21 is a cyclin-dependent kinase inhibitor that blocks cell cycle progression and is involved in transcription, apoptosis, and DNA repair [103]. When double-strand breaks caused by DNA damage are not fully repaired, the activation of ATM and ATR triggers the phosphorylation of p53; the phosphorylated form of p53 then regulates p21-induced cell cycle arrest and subsequently triggers cellular senescence or apoptosis [104].

The active ingredients of ginseng can repair DNA damage by upregulating the activity of DNA glycosylases and sirtuin family members in the DNA damage repair system and inhibiting the cGAS-STING pathway, thereby slowing down the aging process [105,106,107]. DNA glycosylase is one of the key enzymes in the BER pathway, mainly composed of nucleic acid Endonuclease VIII like (NEIL) 1, NEIL2, and NEIL3 proteins [108]. Sirtuins are multifunctional ribosyltransferases with a conserved NAD^+^-dependent catalytic core structural domain. Each family member localizes to a different subcellular compartment and targets a different substrate to control various biological processes, such as DNA damage repair, the maintenance of genomic stability, aging, and tumorigenesis [109].

**Figure 5:**
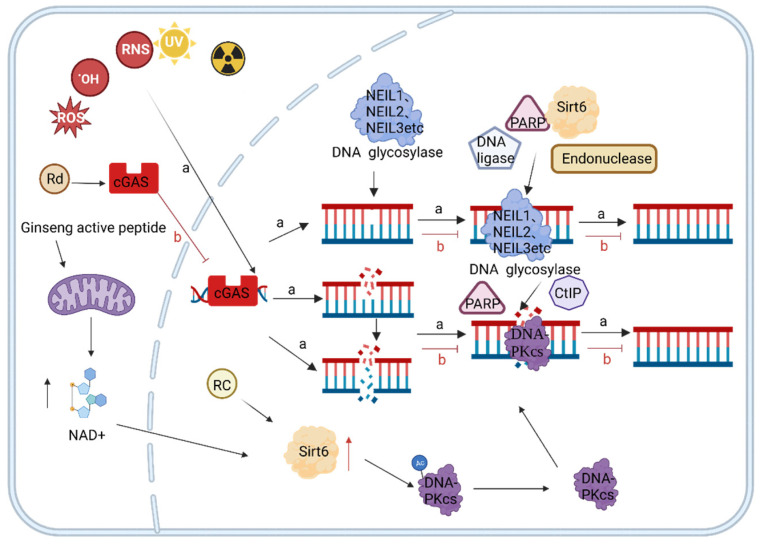
DNA damage repair regulated by ginseng active ingredients. (a) Ginseng active ingredients upregulate the expression of DNA glycosylase and Sirt6 to repair the DNA damage process. (b) Ginseng active ingredients inhibit the CGAS-STING pathway and promote the DNA damage repair process.

DNA glycosylase recognizes damaged DNA after the body’s DNA is attacked and generates an AP site (Figure 5) [110]. Poly(ADP-ribose) polymerase (PARP) family protein factors recognize and bind to AP-nucleic acid endonuclease DNA ligase, facilitating excision repair [62]. In mice, the knockdown of NEIL1 resulted in severe DNA damage [108,111]. Ginsenoside Rd upregulated the expression of NEIL1 and NEIL3 in rat brain cells to reduce DNA damage by promoting the repair function of DNA glycosylases [105]. SIRT6 is a member of the sirtuin family that binds to PARP and repairs damaged DNA by stimulating DNA glycosylase activity [112]. Ginsenoside RC increases the deacetylase activity of SIRT6 and stimulates BER by activating PARP [113].

When a DNA double-strand breakage (DSB) occurs, cellular machinery initiates homologous recombination (HR), NHEJ, and other repair methods. A variety of repair enzymes are involved in the process of DNA homologous recombination repair. DNA-dependent protein kinase, catalytic subunit (DNA-PKcs), and CtIP are indispensable repair enzymes in the DNA break repair process [114,115]. SIRT6 promotes overall DNA repair through the deacetylation of DNA-PKcs and carboxy-terminal binding protein (CtBP)-interacting protein (CtIP) [116]. SIRT6 silencing leads to impaired downstream signaling, and this affects the recruitment of key repair proteins [117]. SIRT6 stimulates DNA repair only in the presence of the coenzyme nicotinamide adenine dinucleotide (NAD^+^) in living cells. Double-strand breaks lead to the activation of PARP1, and excessive PARP1 activation leads to the depletion of NAD^+^ substrate [118]. Ginseng active peptides can increase the expression of SIRT3, SIRT6, and SIRT1 for normal mitochondrial function, increase the content of NAD^+^ for the more efficient repair of sirtuins, and reduce the content of β-galactosidase (a marker of senescence in cells that delays aging) [45].

In addition, cGAS-STING inhibits DNA damage repair [107]. DNA damage activates BER and homologous recombination repair (Figure 4) [119,120]. Upon the occurrence of DNA double-strand breaks, cGAS is dependent on the Y215 tyrosine residue to facilitate its transfer to the nucleus in a dephosphorylated state, while simultaneously avoiding disruption of the nuclear membrane in order to reach the site of DNA damage. This process interferes with the signaling of the homologous recombination pathway, ultimately leading to the inhibition of precise homologous recombination repair [120]. The deactivation of glutathione peroxidase 4 (GPX4) increases lipid peroxidation, inhibits the transfer of STING from the endoplasmic reticulum (ER) to the Golgi apparatus, and reduces the production of inflammatory factors [77]. Ginsenoside Rd inhibited the activation of the CGAS-STING pathway by decreasing GPX4 expression, reducing inflammation, and alleviating acute lung injury to delay aging in mice [78].

### 4.4. Other Anti-Aging Mechanisms of Ginseng

**Figure 6:**
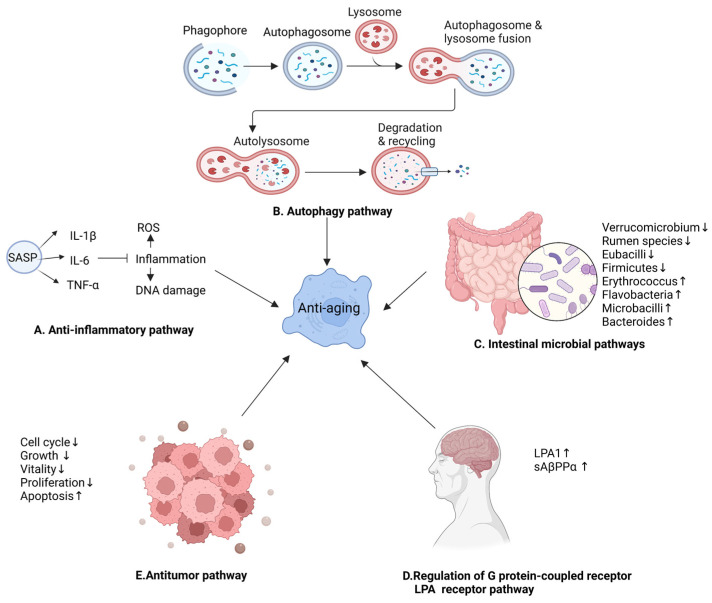
Other anti-aging mechanisms of ginseng. SASP, senescence-associated secretory phenotype.

The active ingredients of ginseng can delay aging through anti-inflammatory mechanisms, the promotion of cellular autophagy, and the regulation of intestinal microbes (Figure 6). Inflammation leads to a decrease in tissue repair and production; this is a major factor associated with aging [121]. The typical immune transcription factor NF-κB is activated in an ATM-dependent manner, suggesting that NF-κB is critical for the expression of pro-inflammatory signaling molecules following DNA damage [122,123]. The NF-κB signaling pathway accelerates the aging process [124]. Pro-inflammatory factors contribute to the development of chronic inflammation in autocrine and paracrine forms [125]. Chronic inflammation secretes cytokines that maintain inflammation and redox stress, exacerbate oxidative damage, and induce ROS, hydrogen peroxide, and hydroxyl radical production, exacerbating DNA damage and accelerating the onset of aging and related diseases [126,127]. The aqueous extracts and active ingredients of ginseng regulate the expression of inflammatory factors and delay aging through the NF-κB signaling pathway. For example, the aqueous extract of Korean red ginseng and ginseng active peptide inhibited the secretion of typical pro-inflammatory factors IL-1β, IL-6, and TNF-α in various organs of aged mice to delay aging [98,99,100]. Ginsenoside Rc targeted TANK-binding kinase 1/interferon regulatory factor-3; inhibited the expression of TNF-α, IL-1, and IFNs; reduced chronic inflammation; and ultimately delayed aging [128,129]. Autophagy refers to the lysosomal degradation and recycling of all types of intracellular components; it is a highly selective cellular clearance pathway associated with the maintenance of cellular and tissue homeostasis [130,131,132]. There are three main types of autophagy: macro-autophagy, micro-autophagy, and chaperone-mediated autophagy [133]. Macro-autophagy involves the formation of autophagosomes with a double-layered membrane structure that engulfs intracellular components [134]. Autophagy mainly includes four processes: the induction of autophagy, the formation of autophagosomes, the transport and fusion of autophagosomes and lysosomes, and degradation and recovery [135]. Impaired autophagy accelerates cellular senescence [3], and autophagy activity decreases with age in different organisms [136]. p62 is an autophagic receptor that plays an important role in the autophagy process [121]. The anti-aging effect of Korean red ginseng is mediated by autophagy [137]. Moreover, it has been reported that, in the model organism, *C. elegans*, ginseng volatile oil delayed aging and prolonged life by increasing the expression of autophagy substrate p62 protein [39]. Rg2 maintains mitochondrial function and delays brain aging by promoting the degradation of p62 [138]. ROS induce the degradation of the extracellular matrix, leading to visible signs of skin aging. The active ingredients in ginseng berries alleviate skin aging through autophagy [139,140].

Ginonin, the active ingredient of ginseng, can delay aging by regulating the LPA receptor in the G protein-coupled receptor (Figure 6). LPA and LPA1 receptors play crucial roles in early brain development [141]. The ginseng active ingredient, gintonin, generates the second messenger, Ca^2+^, via the LPA receptor to activate the Ca^2+^-dependent kinase, receptor, gliotransmitter, and neurotransmitter release, initiating first-order amplification and inducing further intracellular effects as well as intercellular communication [142]. In memory dysfunction, an important condition in neurodegenerative diseases, brain aging affects hippocampal function and induces memory dysfunction. In D-galactose-induced aged murine brains, hippocampal LPA1 receptors are reduced, and gintonin administration increases LPA1 receptor expression in the murine hippocampus [143]. In the age-related neurodegenerative disease, Alzheimer’s disease (AD), gintonin promotes non-amyloid protein, sAβPPα, release via the LPA1 receptor signaling pathway and Ca^2+^-dependent metalloproteinase secretase activation and protein translocation processes [144]. This ultimately prevents A formation and amyloid plaque accumulation in the brains of aged AD model animals and delaying brain aging.

In addition, ginseng active ingredients exhibited good anti-tumor properties. Tumor development is closely related to aging. The ginsenoside active ingredients achieve anti-tumor effects by inhibiting the growth, proliferation, and viability of cancer cells, inducing apoptosis, inhibiting cell cycle, and a series of other pathways [145,146,147,148]. Rh2 achieves anti-tumor activity by inhibiting tumor cell migration, upregulating the pro-apoptotic gene Bax, downregulating the anti-apoptotic gene Bcl-2, and disrupting the HSP90A-CDC37 system in hepatocellular carcinoma cells [149,150]. In addition, the anti-tumor effects of other active components of ginseng have been successively demonstrated. Ginseng polysaccharides achieve anti-tumor effects by altering the gut microbiota and kynurenine/tryptophan ratio, enhancing anti-programmed cell death 1/programmed cell death ligand 1 (anti-PD-1/PD-L1), targeting GPX4, and facilitating macrophage and NK cell activation [151,152,153,154]. Rg3 inhibits thyroid cancer metastasis by suppressing vascular endothelial growth factor-C (VEGF-C) protein expression in PTC cells and VEGF-A protein expression in anaplastic thyroid cancer (ATC) cells [155]. This ultimately reduces melanoma cell proliferation by inhibiting ERK and Akt signaling [156]. Osteosarcoma inhibition is achieved by modulating the Wnt/β-collagen pathway via MMP2, MMP7, and MMP9 downregulation [157].

The active ingredients of ginseng can delay aging by regulating the abundance of intestinal microflora. The human gastrointestinal tract is occupied by various microbial communities that are involved in maintaining the health of the host and in several physiological processes. Changes in the composition of gut microbes influence the onset of various diseases and aging [158,159]. Ginseng exerts powerful anti-aging effects through the modulation of inflammatory pathways and the microbe–gut–brain axis [72]. Korean ginseng inhibits the abundance of inflammation-related microbes, including Verrucomicrobiota, *Ruminococcus*, and Eubacterium, to delay aging and inhibit the death of dopaminergic neurons [160]. Fermented ginseng can regulate the intestinal microbiota of *C. elegans*; improve the composition and structure of the intestinal flora; increase the relative abundance of *Erythrobacter*, *Flavobacterium*, and *Microbacterium*; and prolong the life span of *C. elegans* [161]. Ginsenoside Rh4 reduced the number of Firmicutes and increased the abundance of Bacteroidetes, which resulted in the inhibition of inflammation [162] and a delay in the aging process.

## 5. Discussion

Aging is a permanent process that occurs in all life forms. The anti-aging effect of ginseng has been documented since ancient times. Extracts, oligopeptides, volatile oils, or monomers from ginseng are able to alleviate age-related diseases and delay the aging process [163,164,165,166]. Ginsenosides are the most widely studied active ingredients, and ginsenoside monomers, such as Rg1, Rg3, Rb1, and Rc, have shown strong anti-aging effects and demonstrated good therapeutic effects against neurodegenerative disease, diabetes, skin aging, muscle atrophy, and other age-related diseases [167,168,169,170,171]. The active ingredients of ginseng mainly contribute to aging delay through the following mechanisms: (1) the increase in the expression of antioxidant enzymes to achieve a balance between intracellular oxidation and antioxidation, the mitigation of excessive production of ROS, and the reduction in endogenous DNA damage, which prevent cell cycle arrest and delay aging; and (2) the regulation of the activities of DNA glycosylase and sirtuins in the process of DNA damage repair to ensure that the DNA repair pathway can accurately repair DNA damage caused by various factors associated with aging. 

It is noteworthy that most of the studies examined in this review only assessed longevity, antioxidant enzymes, inflammatory factors, and aging markers in model organisms (such as flies and *C. elegans*). Moreover, research on the activity of galactosidase is also limited to the changes in the levels of proteins associated with the related pathways, and there are few in-depth studies on its role in the molecular mechanism of aging. In addition, studies on the biological activities of ginseng have continued to face problems related to the complexity of its active components, and consequently, the molecular mechanisms underlying the functions of these active components have remained poorly understood. To address these limitations and identify specific mechanisms of action and targets, network pharmacology and CRISPR (clustered regularly interspaced short palindromic repeats) technologies have been used to screen potential drugs from ginseng using knockout mice and cellular models. These innovative methodologies offer promising avenues to increase our understanding of the mechanisms of action associated with the anti-aging effect of ginseng.

## Figures and Tables

**Table 1:** Effect of the purified compounds from ginseng on aging.

Active Ingredient	Biological Effects	In Vivo Model	In Vitro Model	Testing Index	Source
Rg1	Mitigation of DNA damage and antioxidant and anti-aging effects	NRF2^−/−^, C57BL/6 mice intraperitoneally injected with D-galactose (D-gal) for 42 days	D-gal induced primary bone marrow mesenchymal stem cells treated for 24 h	β-Galactosidase, γ-H2AX, p16, p53, p21, IL-6, IL-1β	[40]
	Antioxidant, anti-apoptotic, free radical-scavenging, and anti-inflammatory effects	C57BL/6 intraperitoneally injected with D-gal for 42 days		β-Galactosidase, MDA, SOD, IL-1β, IL-6, TNF-α, p53, p21	[41]
	Antioxidant effect and mitigation of oxidative stress	C57BL/6 intraperitoneally injected with D-gal for 42 days	D-gal stimulation of primary neural stem cells	MDA, SOD, GSH-px, p53, p21, Rb	[42]
	Inhibition of excessive activation of the Wnt/β-linked protein signaling pathway	C57BL/6 mice injected with D-gal for 42 days		ROS, SOD, GSH-px, MDA, c-Myc, GSK-3β, p53, p16, p21	[43]
	Antioxidant and downregulation of aging-related proteins	Sprague Dawley rats injected with D-gal for 42 days		IL-2, IL-6, TNF-α, GSH, SOD, MDA	[44]
Rg3	Downregulation of AKT and regulation of NAD/NADH		Human dermal fibroblasts undergo continuous passaging up to 34–36 generations, allowing them to become senescent cells	SA-β-gal, ROS, sirt1/3/6, NAD/NADH, p21, p53	[45]
Rb1	Regulation of the p53-p21-Cdk2 pathway, cell cycle regulation, and anti-apoptotic effect	C57BL/6 mice fed for 10 months		p53, p21, Cdk2, bax, NF-κB	[46]
Rb2	Induction of autophagy		Human dermal fibroblasts undergo passaging until they become senescent cells in 34 to 36 generations	SA-β-gal, p53, p21, p16, CDK4, p62	[47]
Re	Upregulation of Nrf2/GPx-1/ERK/CERB signaling	Klotho mutant mice		NOX, ROS, GPx, Nrf2, ERK, CERB	[48]

**Table 2:** Effect of the extracts of ginseng on aging.

Active Ingredient	Biological Effects	In Vivo Model	In Vitro Model	Testing Index	Source
Ginsenosides	Anti-apoptotic and antioxidant effect and inhibition of oxidative DNA damage		*Helicobacter pylori* stimulated AGS human gastric epithelial cells (bacteria:cells = 3:1) for 1 h	ROS, Bax/Bcl-2, caspase-3, ATM, Mdm2, ARF	[49]
Total Ginsenoside Aqueous Extract	Inhibition of oxidative stress	*Caenorhabditis elegans* and worms		ROS, NAD+, SIRT1, NRF2	[11]
Ginsenoside aqueous extract	Anti-inflammatory and antioxidant effects		Mir-155-5p inhibitor, human umbilical vein endothelial cells	SA-β-gal, ROS NO, NF-κB, p53, p21	[50]
Ginseng rhamnogalacturonic acid I	Upregulation of DAF-16 and skn-1 activities	*C. elegans*		ROS, Nrf2, DAF-16	[51]
Red ginseng extracts	Anti-inflammatory effect and regulation of antioxidant enzyme activity	C57BL/6 20–21 months		NOS, COX, TNF-α, IL-1β	[52]
Ginseng oligopeptide	Adjustment of the NAD/SIRT1/PGC-1 α pathways to improve mitochondrial function		Embryonic NIH/3T3 fibroblasts treated with H_2_O_2_ for 4 h	γ-H2A.X, ROS, GSH-Px, SOD, MDA	[53]
Ginseng volatile oil	Elimination of free radicals and suppression of oxidation	*C. elegans*		SOD, MDA	[39]
Black ginseng	Inhibition of p53-p21/p16 activation and anti-inflammatory effect	18-month-old C57BL/6 mice	20 Gy γ radiation-induced senescence of primary mouse embryonic fibroblasts and 30 passages of HEK293 cells	SA-β-percentage of gal-positive cells, p53	[54]
Red ginseng	Inhibition of the Akt pathway	36-day-old female *Drosophila melanogaster*		Raf1, ERK, p-ERK, AKT, p-AKT	[55]
Korean ginseng	Regulation of PPAR signaling and antioxidant effect	Dec2^−/−^ mice	HEI-OC1 cells treated with neomycin for 24 h	Dec1, Dec2, Dec25, Il1β, Fabp2	[56]

## References

[1] McHugh D., Gil J. (2018). Senescence and aging: Causes, consequences, and therapeutic avenues. J. Cell Biol..

[2] Herranz N., Gil J. (2018). Mechanisms and functions of cellular senescence. J. Clin. Investig..

[3] Yamamoto-Imoto H., Minami S., Shioda T., Yamashita Y., Sakai S., Maeda S., Yamamoto T., Oki S., Takashima M., Yamamuro T. (2022). Age-associated decline of MondoA drives cellular senescence through impaired autophagy and mitochondrial homeostasis. Cell Rep..

[4] Gorgoulis V., Adams P.D., Alimonti A., Bennett D.C., Bischof O., Bishop C., Campisi J., Collado M., Evangelou K., Ferbeyre G. (2019). Cellular senescence: Defining a path forward. Cell.

[5] Di Micco R., Krizhanovsky V., Baker D., d’Adda di Fagagna F. (2021). Cellular senescence in ageing: From mechanisms to therapeutic opportunities. Nat. Rev. Mol. Cell Biol..

[6] Schumacher B., Pothof J., Vijg J., Hoeijmakers J.H.J. (2021). The central role of DNA damage in the ageing process. Nature.

[7] An M.Y., Lee S.R., Hwang H.J., Yoon J.G., Lee H.J., Cho J.A. (2021). Antioxidant and anti-inflammatory effects of Korean Black ginseng extract through ER stress pathway. Antioxidants.

[8] Sun G., Wang J., Xu X., Zhai L., Li Z., Liu J., Zhao D., Jiang R., Sun L. (2023). *Panax ginseng* Meyer cv. Silvatica phenolic acids protect DNA from oxidative damage by activating Nrf2 to protect HFF-1 cells from UVA-induced photoaging. J. Ethnopharmacol..

[9] Li S., Qi Y., Chen L., Qu D., Li Z., Gao K., Chen J., Sun Y. (2019). Effects of *Panax ginseng* polysaccharides on the gut microbiota in mice with antibiotic-associated diarrhea. Int. J. Biol. Macromol..

[10] Zhao Q., Liu Y., Zhang S., Zhao Y., Wang C., Li K., Jin Z., Qiao J., Liu M. (2022). Studies on the regulation and molecular mechanism of *Panax ginseng* saponins on senescence and related behaviors of *Drosophila melanogaster*. Front. Aging Neurosci..

[11] Wang H., Zhang S., Zhai L., Sun L., Zhao D., Wang Z., Li X. (2021). Ginsenoside extract from ginseng extends lifespan and health span in *Caenorhabditis elegans*. Food Funct..

[12] Yousefzadeh M., Henpita C., Vyas R., Soto-Palma C., Robbins P., Niedernhofer L. (2021). DNA damage-how and why we age?. eLife.

[13] Ciccia A., Elledge S.J. (2010). The DNA damage response: Making it safe to play with knives. Mol. Cell.

[14] He S., Sharpless N.E. (2017). Senescence in health and disease. Cell.

[15] Wiggins K.A., Parry A.J., Cassidy L.D., Humphry M., Webster S.J., Goodall J.C., Narita M., Clarke M.C.H. (2019). IL-1α cleavage by inflammatory caspases of the noncanonical inflammasome controls the senescence-associated secretory phenotype. Aging Cell.

[16] Pignolo R.J., Passos J.F., Khosla S., Tchkonia T., Kirkland J.L. (2020). Reducing senescent cell burden in aging and disease. Trends Mol. Med..

[17] Khosla S., Farr J.N., Tchkonia T., Kirkland J.L. (2020). The role of cellular senescence in ageing and endocrine disease. Nat. Rev. Endocrinol..

[18] Paez-Ribes M., González-Gualda E., Doherty G.J., Muñoz-Espín D. (2019). Targeting senescent cells in translational medicine. EMBO Mol. Med..

[19] Oost W., Talma N., Meilof J.F., Laman J.D. (2018). Targeting senescence to delay progression of multiple sclerosis. J. Mol. Med..

[20] Zhang P., Kishimoto Y., Grammatikakis I., Gottimukkala K., Cutler R.G., Zhang S., Abdelmohsen K., Bohr V.A., Misra Sen J., Gorospe M. (2019). Senolytic therapy alleviates Aβ-associated oligodendrocyte progenitor cell senescence and cognitive deficits in an Alzheimer’s disease model. Nat. Neurosci..

[21] Thompson L.H. (2012). Recognition, signaling, and repair of DNA double-strand breaks produced by ionizing radiation in mammalian cells: The molecular choreography. Mutat. Res..

[22] Nikitaki Z., Holá M., Donà M., Pavlopoulou A., Michalopoulos I., Angelis K.J., Georgakilas A.G., Macovei A., Balestrazzi A. (2018). Integrating plant and animal biology for the search of novel DNA damage biomarkers. Mutat. Res. Rev. Mutat. Res..

[23] Shang D., Sun D., Shi C., Xu J., Shen M., Hu X., Liu H., Tu Z. (2020). Activation of epidermal growth factor receptor signaling mediates cellular senescence induced by certain pro-inflammatory cytokines. Aging Cell.

[24] Sendama W. (2020). The effect of ageing on the resolution of inflammation. Ageing Res. Rev..

[25] Sun L., Wu J., Du F., Chen X., Chen Z.J. (2013). Cyclic GMP-AMP synthase is a cytosolic DNA sensor that activates the type I interferon pathway. Science.

[26] Sun L., Wu D., Ning X., Yang G., Lin Z., Tian M., Zhou Y. (2015). α-Amylase-assisted extraction of polysaccharides from *Panax ginseng*. Int. J. Biol. Macromol..

[27] Zhao B., Lv C., Lu J. (2019). Natural occurring polysaccharides from *Panax ginseng* C. A. Meyer: A review of isolation, structures, and bioactivities. Int. J. Biol. Macromol..

[28] Sun H., Liu F., Sun L., Liu J., Wang M., Chen X., Xu X., Ma R., Feng K., Jiang R. (2016). Proteomic analysis of amino acid metabolism differences between wild and cultivated *Panax ginseng*. J. Ginseng Res..

[29] Qiu Y., Lu X., Pang T., Ma C., Li X., Xu G. (2008). Determination of radix ginseng volatile oils at different ages by comprehensive two-dimensional gas chromatography/time-of-flight mass spectrometry. J. Sep. Sci..

[30] Hyun S.H., Kim S.W., Seo H.W., Youn S.H., Kyung J.S., Lee Y.Y., In G., Park C.K., Han C.K. (2020). Physiological and pharmacological features of the non-saponin components in Korean Red Ginseng. J. Ginseng Res..

[31] Resetar M., Liu X., Herdlinger S., Kunert O., Pferschy-Wenzig E.M., Latkolik S., Steinacher T., Schuster D., Bauer R., Dirsch V.M. (2020). Polyacetylenes from Oplopanax horridus and *Panax ginseng*: Relationship between Structure and PPARγ Activation. J. Nat. Prod..

[32] Jang G.Y., Kim M.Y., Lee Y.J., Li M., Shin Y.S., Lee J., Jeong H.S. (2018). Influence of organic acids and heat treatment on ginsenoside conversion. J. Ginseng Res..

[33] Yoon D., Shin W.C., Oh S.M., Choi B.R., Young Lee D. (2022). Integration of multiplatform metabolomics and multivariate analysis for geographical origin discrimination of *Panax ginseng*. Food Res. Int..

[34] Liu X., Mi X., Wang Z., Zhang M., Hou J., Jiang S., Wang Y., Chen C., Li W. (2020). Ginsenoside Rg3 promotes regression from hepatic fibrosis through reducing inflammation-mediated autophagy signaling pathway. Cell Death Dis..

[35] Xiong X., Huang G., Huang H. (2019). The antioxidant activities of phosphorylated polysaccharide from native ginseng. Int. J. Biol. Macromol..

[36] Chu S.F., Zhang Z., Zhou X., He W.B., Chen C., Luo P., Liu D.D., Ai Q.D., Gong H.F., Wang Z.Z. (2019). Ginsenoside Rg1 protects against ischemic/reperfusion-induced neuronal injury through miR-144/Nrf2/ARE pathway. Acta Pharmacol. Sin..

[37] Long J., Liu X.K., Kang Z.P., Wang M.X., Zhao H.M., Huang J.Q., Xiao Q.P., Liu D.Y., Zhong Y.B. (2022). Ginsenoside Rg1 ameliorated experimental colitis by regulating the balance of M1/M2 macrophage polarization and the homeostasis of intestinal flora. Eur. J. Pharmacol..

[38] Szeto Y.T., Sin Y.S., Pak S.C., Kalle W. (2015). American ginseng tea protects cellular DNA within 2 h from consumption: Results of a pilot study in healthy human volunteers. Int. J. Food Sci. Nutr..

[39] Wang L., Qiao P., Ouyang Z., Li D., Zheng J., Wang G., Wang F. (2022). Ginseng volatile oil prolongs the lifespan and healthspan of *Caenorhabditis elegans*. Biogerontology.

[40] Wang Z., Wang L., Jiang R., Li C., Chen X., Xiao H., Hou J., Hu L., Huang C., Wang Y. (2021). Ginsenoside Rg1 prevents bone marrow mesenchymal stem cell senescence via NRF2 and PI3K/Akt signaling. Free Radic. Biol. Med..

[41] Wang Z.L., Chen L.B., Qiu Z., Chen X.B., Liu Y., Li J., Wang L., Wang Y.P. (2018). Ginsenoside Rg1 ameliorates testicular senescence changes in D-gal-induced aging mice via anti-inflammatory and antioxidative mechanisms. Mol. Med. Rep..

[42] Chen L., Yao H., Chen X., Wang Z., Xiang Y., Xia J., Liu Y., Wang Y. (2018). Ginsenoside Rg1 decreases oxidative stress and down-regulates Akt/mTOR signalling to attenuate cognitive impairment in mice and senescence of neural stem cells induced by D-galactose. Neurochem. Res..

[43] Li J., Cai D., Yao X., Zhang Y., Chen L., Jing P., Wang L., Wang Y. (2016). Protective effect of ginsenoside Rg1 on hematopoietic stem/progenitor cells through attenuating oxidative stress and the Wnt/β-catenin signaling pathway in a mouse model of d-galactose-induced aging. Int. J. Mol. Sci..

[44] Sun J., Zhang L., Zhang J., Ran R., Shao Y., Li J., Jia D., Zhang Y., Zhang M., Wang L. (2018). Protective effects of ginsenoside Rg1 on splenocytes and thymocytes in an aging rat model induced by d-galactose. Int. Immunopharmacol..

[45] Yang K.E., Jang H.J., Hwang I.H., Hong E.M., Lee M.G., Lee S., Jang I.S., Choi J.S. (2020). Stereoisomer-specific ginsenoside 20(S)-Rg3 reverses replicative senescence of human diploid fibroblasts via Akt-mTOR-sirtuin signaling. J. Ginseng Res..

[46] Yu S., Xia H., Guo Y., Qian X., Zou X., Yang H., Yin M., Liu H. (2020). Ginsenoside Rb1 retards aging process by regulating cell cycle, apoptotic pathway and metabolism of aging mice. J. Ethnopharmacol..

[47] Yang K.E., Nam S.B., Jang M., Park J., Lee G.E., Cho Y.Y., Jang B.C., Lee C.J., Choi J.S. (2023). Ginsenoside Rb2 suppresses cellular senescence of human dermal fibroblasts by inducing autophagy. J. Ginseng Res..

[48] Nguyen B.T., Shin E.J., Jeong J.H., Sharma N., Nah S.Y., Ko S.K., Byun J.K., Lee Y., Lei X.G., Kim D.J. (2022). Ginsenoside Re attenuates memory impairments in aged klotho deficient mice via interactive modulations of angiotensin II AT1 receptor, Nrf2 and GPx-1 gene. Free Radic. Biol. Med..

[49] Kang H., Lim J.W., Kim H. (2020). Inhibitory effect of Korean Red ginseng extract on DNA damage response and apoptosis in *Helicobacter pylori*-infected gastric epithelial cells. J. Ginseng Res..

[50] Kim T.H., Kim J.Y., Bae J., Kim Y.M., Won M.H., Ha K.S., Kwon Y.G., Kim Y.M. (2021). Korean Red ginseng prevents endothelial senescence by downregulating the HO-1/NF-κB/miRNA-155-5p/eNOS pathway. J. Ginseng Res..

[51] Wang J., Zhou Y., Yu Y., Wang Y., Xue D., Zhou Y., Li X. (2023). A ginseng-derived rhamnogalacturonan I (RG-I) pectin promotes longevity via TOR signalling in *Caenorhabditis elegans*. Carbohydr. Polym..

[52] Lee Y., Oh S. (2015). Administration of red ginseng ameliorates memory decline in aged mice. J. Ginseng Res..

[53] Zhu N., Xu M.H., Li Y. (2022). Bioactive oligopeptides from ginseng (*Panax ginseng* Meyer) Suppress Oxidative Stress-Induced Senescence in Fibroblasts via NAD+/SIRT1/PGC-1α Signaling Pathway. Nutrients.

[54] Lee S.J., Lee D.Y., O’Connell J.F., Egan J.M., Kim Y. (2022). Black ginseng ameliorates cellular senescence via p53-p21/p16 pathway in aged mice. Biology.

[55] Hou W., Pei J., Wang Y., Zhang J., Zheng H., Cui R. (2020). Anti-ageing effects of red ginseng on female *Drosophila melanogaster*. J. Cell Mol. Med..

[56] Nam Y.H., Jeong S.Y., Kim Y.H., Rodriguez I., Nuankaew W., Bhawal U.K., Hong B.N., Kang T.H. (2021). Anti-aging effects of Korean Red Ginseng (KRG) in differentiated embryo chondrocyte (DEC) knockout mice. J. Ginseng Res..

[57] Tubbs A., Nussenzweig A. (2017). Endogenous DNA damage as a source of genomic instability in cancer. Cell.

[58] Jackson S.P., Bartek J. (2009). The DNA-damage response in human biology and disease. Nature.

[59] He B., Chen D., Zhang X., Yang R., Yang Y., Chen P., Shen Z. (2022). Oxidative stress and ginsenosides: An update on the molecular mechanisms. Oxid. Med. Cell Longev..

[60] Salehi F., Behboudi H., Kavoosi G., Ardestani S.K. (2018). Oxidative DNA damage induced by ROS-modulating agents with the ability to target DNA: A comparison of the biological characteristics of citrus pectin and apple pectin. Sci. Rep..

[61] Srinivas U.S., Tan B.W.Q., Vellayappan B.A., Jeyasekharan A.D. (2019). ROS and the DNA damage response in cancer. Redox Biol..

[62] Carusillo A., Mussolino C.D.N.A. (2020). DNA Damage: From threat to treatment. Cells.

[63] Ali T., Kim T., Rehman S.U., Khan M.S., Amin F.U., Khan M., Ikram M., Kim M.O. (2018). Natural dietary supplementation of anthocyanins via PI3K/Akt/Nrf2/HO-1 pathways mitigate oxidative stress, neurodegeneration, and memory impairment in a mouse model of Alzheimer’s disease. Mol. Neurobiol..

[64] Dai X., Yan X., Wintergerst K.A., Cai L., Keller B.B., Tan Y. (2020). Nrf2: Redox and metabolic regulator of stem cell state and function. Trends Mol. Med..

[65] Sabouny R., Fraunberger E., Geoffrion M., Ng A.C., Baird S.D., Screaton R.A., Milne R., McBride H.M., Shutt T.E. (2017). The Keap1-Nrf2 stress response pathway promotes mitochondrial hyperfusion through degradation of the mitochondrial fission protein Drp1. Antioxid. Redox Signal..

[66] Yamamoto M., Kensler T.W., Motohashi H. (2018). The KEAP1-NRF2 system: A thiol-based sensor-effector apparatus for maintaining redox homeostasis. Physiol. Rev..

[67] Xie Y., Shi X., Sheng K., Han G., Li W., Zhao Q., Jiang B., Feng J., Li J., Gu Y. (2019). PI3K/Akt signaling transduction pathway, erythropoiesis and glycolysis in hypoxia. Mol. Med. Rep..

[68] Morris B.J., Willcox D.C., Donlon T.A., Willcox B.J. (2015). FOXO_3_: A major gene for human longevity—A mini-review. Gerontology.

[69] Wang Y., Sun Z. (2009). Current understanding of klotho. Ageing Res. Rev..

[70] Lim S.W., Shin Y.J., Luo K., Quan Y., Cui S., Ko E.J., Chung B.H., Yang C.W. (2019). Ginseng increases klotho expression by FoxO3-mediated manganese superoxide dismutase in a mouse model of tacrolimus-induced renal injury. Aging..

[71] Katoh M., Katoh M. (2007). Wnt signaling pathway and stem cell signaling network. Clin. Cancer Res..

[72] Hao M., Ding C., Peng X., Chen H., Dong L., Zhang Y., Chen X., Liu W., Luo Y. (2022). Ginseng under forest exerts stronger anti-aging effects compared to garden ginseng probably via regulating PI3K/AKT/mTOR pathway, SIRT1/NF-κB pathway and intestinal flora. Phytomedicine.

[73] Zhang Y., Cai W., Han G., Zhou S., Li J., Chen M., Li H. (2020). Panax notoginseng saponins prevent senescence and inhibit apoptosis by regulating the PI3K-AKT-mTOR pathway in osteoarthritic chondrocytes. Int. J. Mol. Med..

[74] Keijzers G., Bakula D., Scheibye-Knudsen M. (2017). Monogenic diseases of DNA repair. N. Engl. J. Med..

[75] Liu Z., Pan H., Zhang Y., Zheng Z., Xiao W., Hong X., Chen F., Peng X., Pei Y., Rong J. (2022). Ginsenoside-Rg1 attenuates sepsis-induced cardiac dysfunction by modulating mitochondrial damage via the P2X7 receptor-mediated Akt/GSK-3β signaling pathway. J. Biochem. Mol. Toxicol..

[76] Yang Y., Wang L., Zhang C., Guo Y., Li J., Wu C., Jiao J., Zheng H. (2022). Ginsenoside Rg1 improves Alzheimer’s disease by regulating oxidative stress, apoptosis, and neuroinflammation through Wnt/GSK-3β/β-catenin signaling pathway. Chem. Biol. Drug Des..

[77] Cadet J., Wagner J.R. (2013). DNA base damage by reactive oxygen species, oxidizing agents, and UV radiation. Cold Spring Harb. Perspect. Biol..

[78] Chatterjee N., Walker G.C. (2017). Mechanisms of DNA damage, repair, and mutagenesis. Environ. Mol. Mutagen..

[79] Vignard J., Mirey G., Salles B. (2013). Ionizing-radiation induced DNA double-strand breaks: A direct and indirect lighting up. Radiother. Oncol..

[80] de Almeida L.G.N., Thode H., Eslambolchi Y., Chopra S., Young D., Gill S., Devel L., Dufour A. (2022). Matrix Metalloproteinases: From Molecular Mechanisms to Physiology, Pathophysiology, and Pharmacology. Pharmacol. Rev..

[81] Fisher G.J., Quan T., Purohit T., Shao Y., Cho M.K., He T., Varani J., Kang S., Voorhees J.J. (2009). Collagen fragmentation promotes oxidative stress and elevates matrix metalloproteinase-1 in fibroblasts in aged human skin. Am. J. Pathol..

[82] Suto M., Masutomi H., Ishihara K., Masaki H. (2019). A Potato Peel Extract Stimulates Type I Collagen Synthesis via Akt and ERK Signaling in Normal Human Dermal Fibroblasts. Biol. Pharm. Bull..

[83] Shin J.W., Kwon S.H., Choi J.Y., Na J.I., Huh C.H., Choi H.R., Park K.C. (2019). Molecular Mechanisms of Dermal Aging and Antiaging Approaches. Int. J. Mol. Sci..

[84] Liu X.Y., Xiao Y.K., Hwang E., Haeng J.J., Yi T.H. (2019). Antiphotoaging and Antimelanogenesis Properties of Ginsenoside C-Y, a Ginsenoside Rb2 Metabolite from American Ginseng PDD-ginsenoside. Photochem. Photobiol..

[85] Nam J.J., Min J.E., Son M.H., Oh J.H., Kang S. (2017). Ultraviolet- and infrared-induced 11 beta-hydroxysteroid dehydrogenase type 1 activating skin photoaging is inhibited by red ginseng extract containing high concentration of ginsenoside Rg3(S). Photodermatol. Photoimmunol. Photomed..

[86] Hwang E., Park S.Y., Yin C.S., Kim H.T., Kim Y.M., Yi T.H. (2017). Antiaging effects of the mixture of *Panax ginseng* and Crataegus pinnatifida in human dermal fibroblasts and healthy human skin. J. Ginseng Res..

[87] Lee S., Rauch J., Kolch W. (2020). Targeting MAPK Signaling in Cancer: Mechanisms of Drug Resistance and Sensitivity. Int. J. Mol. Sci..

[88] Jiang R., Xu X., Sun Z., Wang F., Ma R., Feng K., Li T., Sun L. (2020). Protective Effects of Ginseng Proteins on Photoaging of Mouse Fibroblasts Induced by UVA. Photochem..

[89] Lee J.O., Kim E., Kim J.H., Hong Y.H., Kim H.G., Jeong D., Kim J., Kim S.H., Park C., Seo D.B. (2018). Antimelanogenesis and skin-protective activities of *Panax ginseng* calyx ethanol extract. J. Ginseng Res..

[90] Liu X.Y., Hwang E., Park B., Ngo H.T.T., Xiao Y.K., Yi T.H. (2018). Ginsenoside C-Mx Isolated from Notoginseng Stem-leaf Ginsenosides Attenuates Ultraviolet B-mediated Photoaging in Human Dermal Fibroblasts. Photochem. Photobiol..

[91] Działo M., Mierziak J., Korzun U., Preisner M., Szopa J., Kulma A. (2016). The Potential of Plant Phenolics in Prevention and Therapy of Skin Disorders. Int. J. Mol. Sci..

[92] Pillaiyar T., Manickam M., Jung S.H. (2017). Recent development of signaling pathways inhibitors of melanogenesis. Cell. Signal..

[93] Dilshat R., Fock V., Kenny C., Gerritsen I., Lasseur R.M.J., Travnickova J., Eichhoff O.M., Cerny P., Möller K., Sigurbjörnsdóttir S. (2021). MITF reprograms the extracellular matrix and focal adhesion in melanoma. eLife.

[94] Lee S.J., Lee W.J., Chang S.E., Lee G.Y. (2015). Antimelanogenic effect of ginsenoside Rg3 through extracellular signal-regulated kinase-mediated inhibition of microphthalmia-associated transcription factor. J. Ginseng Res..

[95] Lee D.Y., Jeong Y.T., Jeong S.C., Lee M.K., Min J.W., Lee J.W., Kim G.S., Lee S.E., Ahn Y.S., Kang H.C. (2015). Melanin Biosynthesis Inhibition Effects of Ginsenoside Rb2 Isolated from *Panax ginseng* Berry. J. Microbiol. Biotechnol..

[96] Lee J.E., Park J.I., Myung C.H., Hwang J.S. (2017). Inhibitory effects of ginsenosides on basic fibroblast growth factor-induced melanocyte proliferation. J. Ginseng Res..

[97] Aitken G.R., Henderson J.R., Chang S.C., McNeil C.J., Birch-Machin M.A. (2007). Direct monitoring of UV-induced free radical generation in HaCaT keratinocytes. Clin. Exp. Dermatol..

[98] Oh S.J., Oh Y., Ryu I.W., Kim K., Lim C.J. (2016). Protective properties of ginsenoside Rb3 against UV-B radiation-induced oxidative stress in HaCaT keratinocytes. Biosci. Biotechnol. Biochem..

[99] Hernández Borrero L.J., El-Deiry W.S. (2021). Tumor suppressor p53: Biology, signaling pathways, and therapeutic targeting. Biochim. Biophys. Acta-Rev. Cancer..

[100] You L., Cho J.Y. (2021). The regulatory role of Korean ginseng in skin cells. J. Ginseng Res..

[101] McKinnon P.J. (2017). Genome integrity and disease prevention in the nervous system. Genes. Dev..

[102] Chow H.M., Herrup K. (2015). Genomic integrity and the ageing brain. Nat. Rev. Neurosci..

[103] Ticli G., Cazzalini O., Stivala L.A., Prosperi E. (2022). Revisiting the function of p21CDKN1A in DNA repair: The influence of protein interactions and stability. Int. J. Mol. Sci..

[104] Chen J. (2016). The cell-cycle arrest and apoptotic functions of p53 in tumor initiation and progression. Cold Spring Harb. Perspect. Med..

[105] Yang L.X., Zhang X., Zhao G. (2016). Ginsenoside Rd attenuates DNA damage by increasing expression of DNA glycosylase endonuclease VIII-like proteins after focal cerebral ischemia. Chin. Med. J..

[106] Pao P.C., Patnaik D., Watson L.A., Gao F., Pan L., Wang J., Adaikkan C., Penney J., Cam H.P., Huang W.C. (2020). HDAC1 modulates OGG1-initiated oxidative DNA damage repair in the aging brain and Alzheimer’s disease. Nat. Commun..

[107] Jiang H., Xue X., Panda S., Kawale A., Hooy R.M., Liang F., Sohn J., Sung P., Gekara N.O. (2019). Chromatin-bound cGAS is an inhibitor of DNA repair and hence accelerates genome destabilization and cell death. EMBO J..

[108] Sykora P., Misiak M., Wang Y., Ghosh S., Leandro G.S., Liu D., Tian J., Baptiste B.A., Cong W.N., Brenerman B.M. (2015). DNA polymerase β deficiency leads to neurodegeneration and exacerbates Alzheimer disease phenotypes. Nucleic Acids Res..

[109] Morigi M., Perico L., Benigni A. (2018). Sirtuins in renal health and disease. J. Am. Soc. Nephrol..

[110] Krokan H.E., Bjørås M. (2013). Base excision repair. Cold Spring Harb. Perspect. Biol..

[111] Chakraborty A., Wakamiya M., Venkova-Canova T., Pandita R.K., Aguilera-Aguirre L., Sarker A.H., Singh D.K., Hosoki K., Wood T.G., Sharma G. (2015). Neil2-null mice accumulate oxidized DNA bases in the transcriptionally active sequences of the genome and are susceptible to innate inflammation. J. Biol. Chem..

[112] Mao Z., Hine C., Tian X., Van Meter M., Au M., Vaidya A., Seluanov A., Gorbunova V. (2011). SIRT6 promotes DNA repair under stress by activating PARP1. Science.

[113] Pan Z., Guo J., Tang K., Chen Y., Gong X., Chen Y., Zhong Y., Xiao X., Duan S., Cui T. (2022). Ginsenoside Rc modulates SIRT6-NRF2 interaction to alleviate alcoholic liver disease. J. Agric. Food Chem..

[114] Chang H.H.Y., Pannunzio N.R., Adachi N., Lieber M.R. (2017). Non-homologous DNA end joining and alternative pathways to double-strand break repair. Nat. Rev. Mol. Cell Biol..

[115] Reginato G., Cannavo E., Cejka P. (2017). Physiological protein blocks direct the Mre11-Rad50-Xrs2 and Sae2 nuclease complex to initiate DNA end resection. Genes. Dev..

[116] Cagnetta A., Soncini D., Orecchioni S., Talarico G., Minetto P., Guolo F., Retali V., Colombo N., Carminati E., Clavio M. (2018). Depletion of SIRT6 enzymatic activity increases acute myeloid leukemia cells’ vulnerability to DNA-damaging agents. Haematologica..

[117] Chen W., Liu N., Zhang H., Zhang H., Qiao J., Jia W., Zhu S., Mao Z., Kang J. (2017). Sirt6 promotes DNA end joining in iPSCs derived from old mice. Cell Rep..

[118] Ronowska A., Szutowicz A., Bielarczyk H., Gul-Hinc S., Klimaszewska-Łata J., Dyś A., Zyśk M., Jankowska-Kulawy A. (2018). The regulatory effects of acetyl-CoA distribution in the healthy and diseased brain. Front. Cell Neurosci..

[119] Tang S., Stokasimov E., Cui Y., Pellman D. (2022). Breakage of cytoplasmic chromosomes by pathological DNA base excision repair. Nature.

[120] Liu H., Zhang H., Wu X., Ma D., Wu J., Wang L., Jiang Y., Fei Y., Zhu C., Tan R. (2018). Nuclear cGAS suppresses DNA repair and promotes tumorigenesis. Nature.

[121] Aliper A.M., Bozdaganyan M.E., Orekhov P.S., Zhavoronkov A., Osipov A.N. (2019). Replicative and radiation-induced aging: A comparison of gene expression profiles. Aging.

[122] Chen Y.L., Tang C., Zhang M.Y., Huang W.L., Xu Y., Sun H.Y., Yang F., Song L.L., Wang H., Mu L.L. (2019). Blocking ATM-dependent NF-κB pathway overcomes niche protection and improves chemotherapy response in acute lymphoblastic leukemia. Leukemia.

[123] Dunphy G., Flannery S.M., Almine J.F., Connolly D.J., Paulus C., Jønsson K.L., Jakobsen M.R., Nevels M.M., Bowie A.G., Unterholzner L. (2018). Non-canonical activation of the DNA sensing adaptor STING by ATM and IFI16 mediates NF-κB signaling after nuclear DNA damage. Mol. Cell.

[124] Tian Y., Li H., Qiu T., Dai J., Zhang Y., Chen J., Cai H. (2019). Loss of PTEN induces lung fibrosis via alveolar epithelial cell senescence depending on NF-κB activation. Aging Cell.

[125] Uyar B., Palmer D., Kowald A., Murua Escobar H., Barrantes I., Möller S., Akalin A., Fuellen G. (2020). Single-cell analyses of aging, inflammation and senescence. Ageing Res. Rev..

[126] Bektas A., Schurman S.H., Sen R., Ferrucci L. (2018). Aging, inflammation and the environment. Exp. Gerontol..

[127] Pezone A., Olivieri F., Napoli M.V., Procopio A., Avvedimento E.V., Gabrielli A. (2023). Inflammation and DNA damage: Cause, effect or both. Nat. Rev. Rheumatol..

[128] Yu T., Yang Y., Kwak Y.S., Song G.G., Kim M.Y., Rhee M.H., Cho J.Y. (2017). Ginsenoside Rc from *Panax ginseng* exerts anti-inflammatory activity by targeting TANK-binding kinase 1/interferon regulatory factor-3 and p38/ATF-2. J. Ginseng Res..

[129] Yu T., Rhee M.H., Lee J., Kim S.H., Yang Y., Kim H.G., Kim Y., Kim C., Kwak Y.S., Kim J.H. (2016). Ginsenoside Rc from Korean Red ginseng (*Panax ginseng C.A. Meyer*) Attenuates inflammatory Symptoms of Gastritis, Hepatitis and Arthritis. Am. J. Chin. Med..

[130] Kaushik S., Tasset I., Arias E., Pampliega O., Wong E., Martinez-Vicente M., Cuervo A.M. (2021). Autophagy and the hallmarks of aging. Ageing Res. Rev..

[131] Kirkin V. (2020). History of the selective autophagy research: How did it begin and where does it stand today?. J. Mol. Biol..

[132] Gatica D., Lahiri V., Klionsky D.J. (2018). Cargo recognition and degradation by selective autophagy. Nat. Cell Biol..

[133] Eckhart L., Tschachler E., Gruber F. (2019). Autophagic control of skin aging. Front. Cell Dev. Biol..

[134] Galluzzi L., Green D.R. (2019). Autophagy-independent functions of the autophagy machinery. Cell.

[135] Li W., He P., Huang Y., Li Y.F., Lu J., Li M., Kurihara H., Luo Z., Meng T., Onishi M. (2021). Selective autophagy of intracellular organelles: Recent research advances. Theranostics.

[136] Leidal A.M., Levine B., Debnath J. (2018). Autophagy and the cell biology of age-related disease. Nat. Cell Biol..

[137] Kim J.K., Shin K.K., Kim H., Hong Y.H., Choi W., Kwak Y.S., Han C.K., Hyun S.H., Cho J.Y. (2021). Korean Red Ginseng exerts anti-inflammatory and autophagy-promoting activities in aged mice. J. Ginseng Res..

[138] Zhang J.J., Chen K.C., Zhou Y., Wei H., Qi M.H., Wang Z., Zheng Y.N., Chen R.X., Liu S., Li W. (2022). Evaluating the effects of mitochondrial autophagy flux on ginsenoside Rg2 for delaying D-galactose induced brain aging in mice. Phytomedicine Int. J. Phytother. Phytopharm..

[139] Kim J., Cho S.Y., Kim S.H., Cho D., Kim S., Park C.W., Shimizu T., Cho J.Y., Seo D.B., Shin S.S. (2017). Effects of Korean ginseng berry on skin antipigmentation and antiaging via FoxO3a activation. J. Ginseng Res..

[140] Choi W., Kim H.S., Park S.H., Kim D., Hong Y.D., Kim J.H., Cho J.Y. (2022). Syringaresinol derived from *Panax ginseng* berry attenuates oxidative stress-induced skin aging via autophagy. J. Ginseng Res..

[141] Cho Y.J., Choi S.H., Lee R., Hwang H., Rhim H., Cho I.H., Kim H.C., Lee J.I., Hwang S.H., Nah S.Y. (2020). Ginseng Gintonin Contains Ligands for GPR40 and GPR55. Molecules.

[142] Choi S.H., Kim H.J., Cho H.J., Park S.D., Lee N.E., Hwang S.H., Cho I.H., Hwang H., Rhim H., Kim H.C. (2019). Gintonin, a Ginseng-Derived Exogenous Lysophosphatidic Acid Receptor Ligand, Protects Astrocytes from Hypoxic and Re-oxygenation Stresses Through Stimulation of Astrocytic Glycogenolysis. Mol. Neurobiol..

[143] Nam S.M., Hwang H., Seo M., Chang B.J., Kim H.J., Choi S.H., Rhim H., Kim H.C., Cho I.H., Nah S.Y. (2018). Gintonin Attenuates D-Galactose-Induced Hippocampal Senescence by Improving Long-Term Hippocampal Potentiation, Neurogenesis, and Cognitive Functions. Gerontology.

[144] Hwang S.H., Shin E.J., Shin T.J., Lee B.H., Choi S.H., Kang J., Kim H.J., Kwon S.H., Jang C.G., Lee J.H. (2012). Gintonin, a ginseng-derived lysophosphatidic acid receptor ligand, attenuates Alzheimer’s disease-related neuropathies: Involvement of non-amyloidogenic processing. JAD.

[145] Gao Q., Zheng J. (2018). Ginsenoside Rh2 inhibits prostate cancer cell growth through suppression of microRNA-4295 that activates CDKN1A. Cell Prolif..

[146] Zhang Q., Hong B., Wu S., Niu T. (2015). Inhibition of prostatic cancer growth by ginsenoside Rh2. Tumor. Biol..

[147] Tang Y., Chen J., Li J., Zheng Y., Zhong X., Huang S., Chen B., Peng B., Zou X., Chen X. (2021). Pristimerin synergistically sensitizes conditionally reprogrammed patient derived-primary hepatocellular carcinoma cells to sorafenib through endoplasmic reticulum stress and ROS generation by modulating Akt/FoxO1/p27(kip1) signaling pathway. Phytomedicine.

[148] Xiaodan S., Ying C. (2022). Role of ginsenoside Rh2 in tumor therapy and tumor microenvironment immunomodulation. Biomed. Pharmacother..

[149] Lev-Ari S., Starr A.N., Vexler A., Kalich-Philosoph L., Yoo H.S., Kwon K.R., Yadgar M., Bondar E., Bar-Shai A., Volovitz I. (2021). Rh2-enriched Korean ginseng (Ginseng Rh^2+^) inhibits tumor growth and development of metastasis of non-small cell lung cancer. Food Funct..

[150] Chen C., Wang Y.S., Zhang E.T., Li G.A., Liu W.Y., Li Y., Jin Y.H. (2021). (20S) Ginsenoside Rh2 Exerts Its Anti-Tumor Effect by Disrupting the HSP90A-Cdc37 System in Human Liver Cancer Cells. Int. J. Mol. Sci..

[151] Huang J., Liu D., Wang Y., Liu L., Li J., Yuan J., Jiang Z., Jiang Z., Hsiao W.W., Liu H. (2022). Ginseng polysaccharides alter the gut microbiota and kynurenine/tryptophan ratio, potentiating the antitumour effect of antiprogrammed cell death 1/programmed cell death ligand 1 (anti-PD-1/PD-L1) immunotherapy. Gut.

[152] Zhai F.G., Liang Q.C., Wu Y.Y., Liu J.Q., Liu J.W. (2022). Red ginseng polysaccharide exhibits anticancer activity through GPX4 downregulation-induced ferroptosis. Pharm. Biol..

[153] Shin M.S., Hwang S.H., Yoon T.J., Kim S.H., Shin K.S. (2017). Polysaccharides from ginseng leaves inhibit tumor metastasis via macrophage and NK cell activation. Int. J. Biol. Macromol..

[154] Lee D.Y., Park C.W., Lee S.J., Park H.R., Seo D.B., Park J.Y., Park J., Shin K.S. (2019). Immunostimulating and Antimetastatic Effects of Polysaccharides Purified from Ginseng Berry. Am. J. Chin. Med..

[155] Wu W., Zhou Q., Zhao W., Gong Y., Su A., Liu F., Liu Y., Li Z., Zhu J. (2018). Ginsenoside Rg3 Inhibition of Thyroid Cancer Metastasis Is Associated with Alternation of Actin Skeleton. J. Med. Food..

[156] Meng L., Ji R., Dong X., Xu X., Xin Y., Jiang X. (2019). Antitumor activity of ginsenoside Rg3 in melanoma through downregulation of the ERK and Akt pathways. Int. J. Oncol..

[157] Mao X., Jin Y., Feng T., Wang H., Liu D., Zhou Z., Yan Q., Yang H., Yang J., Yang J. (2020). Ginsenoside Rg3 Inhibits the Growth of Osteosarcoma and Attenuates Metastasis through the Wnt/β-Catenin and EMT Signaling Pathway. eCAM.

[158] Carmody R.N., Gerber G.K., Luevano J.M., Gatti D.M., Somes L., Svenson K.L., Turnbaugh P.J. (2015). Diet dominates host genotype in shaping the murine gut microbiota. Cell Host Microbe.

[159] Conway J., Duggal A.N. (2021). Ageing of the gut microbiome: Potential influences on immune senescence and inflammageing. Ageing Res. Rev..

[160] Jeon H., Bae C.H., Lee Y., Kim H.Y., Kim S. (2021). Korean red ginseng suppresses 1-methyl-4-phenyl-1,2,3,6-tetrahydropyridine-induced inflammation in the substantia nigra and colon. Brain Behav. Immun..

[161] Xu H.Y., Li Q.C., Zhou W.J., Zhang H.B., Chen Z.X., Peng N., Gong S.Y., Liu B., Zeng F. (2023). Anti-oxidative and anti-aging effects of probiotic fermented ginseng by modulating gut microbiota and metabolites in *Caenorhabditis elegans*. Plant Foods Hum. Nutr..

[162] Bai X., Fu R., Duan Z., Liu Y., Zhu C., Fan D. (2021). Ginsenoside Rh4 alleviates antibiotic-induced intestinal inflammation by regulating the TLR4-MyD88-MAPK pathway and gut microbiota composition. Food Funct..

[163] Fan W., Huang Y., Zheng H., Li S., Li Z., Yuan L., Cheng X., He C., Sun J. (2020). Ginsenosides for the treatment of metabolic syndrome and cardiovascular diseases: Pharmacology and mechanisms. Biomed. Pharmacother..

[164] Zheng Q., Bao X.Y., Zhu P.C., Tong Q., Zheng G.Q., Wang Y. (2017). Ginsenoside Rb1 for myocardial ischemia/reperfusion injury: Preclinical evidence and possible mechanisms. Oxid. Med. Cell Longev..

[165] Dai X., Zeng G., Hong L., Ye Q., Chen X., Zhang J. (2022). Ginsenoside Rg1 and astaxanthin act on the hypothalamus to protect female mice against reproductive aging. Chin. Med. J..

[166] Li Y., Chen C., Li S., Jiang C. (2019). Ginsenoside Rf relieves mechanical hypersensitivity, depression-like behavior, and inflammatory reactions in chronic constriction injury rats. Phytother. Res..

[167] Gao Y., Li J., Chu S., Zhang Z., Chen N., Li L., Zhang L. (2020). Ginsenoside Rg1 protects mice against streptozotocin-induced type 1 diabetic by modulating the NLRP3 and Keap1/Nrf2/HO-1 pathways. Eur. J. Pharmacol..

[168] Zhou T., Zu G., Zhang X., Wang X., Li S., Gong X., Liang Z., Zhao J. (2016). Neuroprotective effects of ginsenoside Rg1 through the Wnt/β-catenin signaling pathway in both in vivo and in vitro models of Parkinson’s disease. Neuropharmacology.

[169] Xie L., Zhai R., Chen T., Gao C., Xue R., Wang N., Wang J., Xu Y., Gui D. (2020). Panax Notoginseng Ameliorates podocyte EMT by targeting the Wnt/β-catenin signaling pathway in STZ-induced diabetic rats. Drug Des. Dev. Ther..

[170] Ryu S., Jeon H., Kim H.Y., Koo S., Kim S. (2020). Korean red ginseng promotes hippocampal neurogenesis in mice. Neural Regen. Res..

[171] Cho D.E., Choi G.M., Lee Y.S., Hong J.P., Yeom M., Lee B., Hahm D.H. (2022). Long-term administration of red ginseng non-saponin fraction rescues the loss of skeletal muscle mass and strength associated with aging in mice. J. Ginseng Res..

